# Giving Drugs a Second Chance: Overcoming Regulatory and Financial Hurdles in Repurposing Approved Drugs As Cancer Therapeutics

**DOI:** 10.3389/fonc.2017.00273

**Published:** 2017-11-14

**Authors:** J. Javier Hernandez, Michael Pryszlak, Lindsay Smith, Connor Yanchus, Naheed Kurji, Vijay M. Shahani, Steven V. Molinski

**Affiliations:** ^1^Department of Molecular Genetics, Faculty of Medicine, University of Toronto, Toronto, ON, Canada; ^2^Lunenfeld-Tanenbaum Research Institute, Toronto, ON, Canada; ^3^The Hospital for Sick Children, Toronto, ON, Canada; ^4^Cyclica Inc., Toronto, ON, Canada

**Keywords:** drug repurposing, funding opportunities, *in silico* predictions, *in vitro* validation, intellectual property laws, oncology indications, regulatory approval

## Abstract

The repositioning or “repurposing” of existing therapies for alternative disease indications is an attractive approach that can save significant investments of time and money during drug development. For cancer indications, the primary goal of repurposed therapies is on efficacy, with less restriction on safety due to the immediate need to treat this patient population. This report provides a high-level overview of how drug developers pursuing repurposed assets have previously navigated funding efforts, regulatory affairs, and intellectual property laws to commercialize these “new” medicines in oncology. This article provides insight into funding programs (e.g., government grants and philanthropic organizations) that academic and corporate initiatives can leverage to repurpose drugs for cancer. In addition, we highlight previous examples where secondary uses of existing, Food and Drug Administration- or European Medicines Agency-approved therapies have been predicted *in silico* and successfully validated *in vitro* and/or *in vivo* (i.e., animal models and human clinical trials) for certain oncology indications. Finally, we describe the strategies that the pharmaceutical industry has previously employed to navigate regulatory considerations and successfully commercialize their drug products. These factors must be carefully considered when repurposing existing drugs for cancer to best benefit patients and drug developers alike.

## Introduction

Drug repurposing is gaining popularity as an approach to develop new medicines. In fact, this strategy of using existing therapeutics for new indications has demonstrated success through previous observational studies and serendipity, such as sildenafil (Viagra), a phosphodiesterase inhibitor initially developed to treat angina and now repurposed as a medication for erectile dysfunction, as well as metformin (Glucophage), a common diabetes medication that is now the active chemical in 100+ ongoing Phase II and Phase III clinical trials as a cancer therapeutic (1). Ideal candidates for drug repurposing are entities that have undergone clinical trials and have been unsuccessful for reasons other than safety (i.e., failed efficacy milestones). Since these drugs have already been deemed safe, development costs are reduced when commencing trials for a new indication. For example, repurposing of the emergency contraceptive, mifepristone, for Cushing’s syndrome required a cohort of less than 30 patients to test its efficacy, whereas a clinical trial^1^ for the same indication evaluating the safety and efficacy of a new chemical entity, levoketoconazole, required ~90 individuals (2, 3).

Finding a new use for an “old” drug holds many appeals. Typically, the safety, efficacy, and toxicity of an existing drug have been extensively studied, and thus, robust data have already been collected toward gaining approval by the United States (US) Food and Drug Administration (FDA) and/or the European Medicines Agency (EMA) for a specific indication. Since data already exist, repurposing saves time and money, which provides hope to patients with rare cancers whose conditions are cost prohibitive for *de novo* development (4). Further, repurposed drugs are generally approved sooner (3–12 years) and at reduced (50–60%) cost (5, 6). In addition, while ~10% of new drug applications gain market approval, approximately 30% of repurposed drugs are approved, giving companies a market-driven incentive to repurpose existing assets (5).

In the context of cancer, rare or terminal oncological manifestations afford less restriction on safety due to the dire need of novel therapies (7, 8). In addition, cancer is a multistage illness with intervention possible during initiation, rapid heterogenous growth, metastasis, and/or recurrence. These features suggest that cancer-focused drug repurposing would be mutually beneficial for patients and pharmaceutical companies alike, with the following sections providing an overview of current opportunities and potential challenges when venturing into this field.

## Overview of Drug Repurposing Funding Initiatives for Cancer

The relative deficit of dedicated funding opportunities for both academic and corporate drug developers reflects the immaturity of drug repurposing initiatives. Academic labs have successfully integrated repurposing initiatives into long-term research grants offered by governmental agencies and patient advocacy groups. These funding opportunities are termed non-dilutive, as the institution receiving the capital does not yield their equity or “dilute” their shares. While companies typically pursue dilutive funding sources, where they leverage investment from venture capitalists and partnerships with larger pharmaceutical companies in exchange for equity in the company. Although these approaches have facilitated preclinical and clinical research, these non-dilutive grants and dilutive investments are highly competitive and scarce, and therefore do not provide sufficient funding to sustain global efforts. Pharmaceutical companies do not offer funding opportunities for such research, as licensing and patent protection obstacles leave limited financial incentives for repurposing generic drugs (9). However, new funding programs specific for drug repurposing initiatives have been established by both governmental and philanthropic organizations to fuel this industry.

## Governmental Granting Agencies

Governmental financial support for drug repurposing began in the US with creation of the National Centre for Advancing Translational Sciences (NCATS)^2^ within the National Institutes of Health in 2012. NCATS facilitates the development of technology to aid in the generation and implementation of novel therapeutics. Thus, NCATS has dedicated resources for drug repurposing efforts, although not explicitly focused on cancer-based projects. Further, NCATS offers research grants for various stages of drug repurposing, from early *in silico* predictions to late-stage clinical trials. Many additional funding agencies exist, such as the National Cancer Institute (US) and the Ontario Institute for Cancer Research (Canada); however, these agencies typically do not provide repurposing-centric grants/subsidies for academics or industrial partners.

In addition, the Canadian Institutes of Health Research (CIHR) partnered with Muscular Dystrophy Canada to develop two specific grants to support drug repurposing programs: the E-Rare 3 joint translational call (JTC) and the North American Re:Rare (NAR:R).^3^ The JTC offers funding for Phase Ib or IIa clinical trials and is cofunded with European partners, whereas the NAR:R was developed by partnering with philanthropic organizations—Cures Within Reach (CWR), the Mindset Foundation, and Mitacs—and offers funding for proof-of-principle research. Unlike NCATS, CIHR has yet to establish funding opportunities for basic science research dedicated to drug repurposing, let alone repurposing efforts toward cancer therapeutics.

## Philanthropic Organizations

Drug repurposing research is also supported by philanthropic organizations. CWR currently funds several clinical trials in progress; for example, repurposing mebendazole (an antiparasitic drug) for brain cancer (i.e., medulloblastoma and glioblastoma).^4^ Other organizations with a similar mandate exist, including the Belgium-based Anticancer Fund, which has dedicated funding to repurpose drug cocktails to treat cancer (e.g., metzolimos, metronomic cyclophosphamide, and methotrexate, combined with sirolimus and zoledronic acid to treat osteosarcoma; clarithromycin, pioglitazone, and treosulfan for non-small cell lung cancer).^5^ In addition, a United Kingdom-based organization, Findacure, launched their first grant opportunity in March 2017 to support drug repurposing research for rare diseases. Some organizations (e.g., Stem Cell Network and Global Cures) either independently fund a repurposing project to completion or co-funds applicants together with governmental agencies or patient advocacy groups; this type of funding requires short-term and highly focused milestones, and therefore is limited in scope. While many agencies recognize the advantages of drug repurposing, most funding opportunities only support late-stage programs, leaving preclinical studies to rely on basic research grants (Table 1). However, as the field continues to deliver effective and cost-efficient therapeutics to patients, more oncology-specific funding opportunities will likely emerge.

**Table 1:** Non-dilutive funding opportunities for drug repurposing initiatives.

Research type	Name of funding opportunity	Source	Maximum available funds (USD)	Duration (years)	Geographical eligibility	Previous repurposing for cancer
Basic science	Disease Team Research Program	SCN^a^	$500,000	–	North America	N
Basic science or preclinical	RFA-TR (UG3)	NCATS^b^	$300,000/year	4	North America	Y
Preclinical	Linking Clinical Trials to Drug Discovery and Repurposing Award	UNM-HSC^c^	$50,000	1	North America	N
Preclinical or clinical	RFP Invitation	CWR^d^	$250,000	3	Global	Y
Preclinical or clinical	Therapeutic Pipeline Program	MJF^e^	$400,000	–	North America	N
Clinical	RFA-TR (UH3)	NCATS^f^	$500,000/year	4	North America	Y
Clinical	Rare Repurposing Open Call	FC^g^	$100,000/year	3	Global	N
Clinical	JTC	CIHR^h^	$377,900/year	3	Canada	N
Clinical	NAR:R	CIHR/CWR^i^	$37,790/year	3	North America	N
Clinical	Various	ACF^j^	–	–	Global	Y
Clinical	Various	GC^k^	–	–	Global	Y

*SCN, Stem Cell Network; NCATS, National Center for Advancing Translational Sciences; UNM-HSC, University of New Mexico Health Sciences Center; CWR, Cures Within Reach; MJF, Michael J. Fox Foundation; FC, Findacure; CIHR, Canadian Institute for Health Research; ACF, Anticancer Fund; GC, Global Cures; JTC, joint translational call*.

*^a^https://stemcellnetwork.ca/funding/*.

*^b^https://grants.nih.gov/grants/guide/rfa-files/RFA-TR-17-002.html#_Section_II._Award_1*.

*^c^https://hsc.unm.edu/research/ctsc/pilot-funding/clinical-trials-to-drug-discovery--repurposing-award/index.html*.

*^d^https://app.cureaccelerator.org/rfp*.

*^e^https://www.michaeljfox.org/research/grant-detail.php?id=28*.

*^f^https://grants.nih.gov/grants/guide/rfa-files/RFA-TR-17-002.html#_Section_II._Award_1*.

*^g^http://www.findacure.org.uk/open-call*.

*^h^http://www.erare.eu/joint-call/e-rare-3-call-proposals-2016-jtc-2016-clinical-research-new-therapeutic-uses-already-0*.

*^i^https://app.cureaccelerator.org/home*.

*^j^http://www.anticancerfund.org/projects/currently-ongoing*.

*^k^https://www.global-cures.org/program/fund*.

## Preclinical Validation of Drug Repurposing Candidates in Oncology

### *In Silico* Discovery and *In Vitro* Validation

With the ever-increasing wealth of public and private data generated through *in vitro, in vivo* and clinical studies, it is becoming increasingly common to leverage biological multi-systems-level big data as well as *in silico* methods to identify novel therapeutics (10, 11). Importantly, two major strategies take either a gene expression or drug-target approach. For example, the Connectivity Map (CMap) is a repository of gene expression profiles derived from human cells treated with various bioactive small molecules (12). Studying a drug’s ability to alter expression profiles in cancer cells allows for inferences to be made about mechanism-of-action. This approach has led to the discovery of antitumor properties of trifluoperazine, an antidepressant previously approved for schizophrenia (13). Encouragingly, trifluoperazine was validated both *in vitro* and *in vivo* and has even demonstrated synergy with the current standard of care (i.e., gefitinib) (14). Another group used CMap to identify 28 compounds exhibiting *in vitro* activity against hepatocellular carcinoma, two of which (chlorpromazine, trifluoperazine) have been validated *in vivo* (15), as well as several other phenothiazines that demonstrate efficacy in tamoxifen-resistant breast cancer cell lines (16). Other gene expression based algorithms also exist, such as the Differentially Expressed Gene Signatures—Inhibitors (DeSigN); this tool has recently identified bosutinib, a kinase inhibitor currently used in leukemia treatment, which proved to be effective *in vitro* on oral squamous cell carcinoma cell lines, further highlighting the power of such approaches (17).

*In silico* drug-target approaches have also been successful. For example, Ke and colleagues identified six compounds that inhibited fibroblast growth factor receptor 3, a biomarker of bladder cancer; two of these were validated *in vitro*, while another demonstrated efficacy in a xenograft mouse model (18). In addition, cyclin-dependent kinase 2 (CDK2) has emerged as a biomarker of various cancers, making it another attractive drug target (19–22). Accordingly, Shi and colleagues have developed protein-ligand docking software which predicted adapalene and fluspirilene as CDK2 inhibitors in colon and liver cancer, respectively; these predictions were validated *in vitro* and *in vivo* (23–25). Given these successes, additional research programs are using computational tools to facilitate repurposing of existing therapies for cancer indications.

Multidisciplinary methods have also been successful. Huang and colleagues combined protein–protein interaction networks with CMap to identify 11 potential drugs to treat non-small cell lung cancer, five of which inhibited cancer cell growth *in vitro* (23). In addition, Lan and colleagues applied machine-learning strategies to complement systems biology data to enrich for true positives; the authors identified 87 potential therapies for nasopharyngeal carcinoma, where over half had been previously described as having anticancer properties (26, 27). These examples underscore the importance of validation to continually improve computational algorithms with empirical evidence.

While *in silico* successes have been highlighted, not all candidates go on to be validated. Potential challenges include would-be true positive hits initially scoring low and being discarded. Encouragingly, since many compounds act as pro-drugs and must be activated *in vivo* [e.g., tamoxifen (28)], it is plausible that certain anticancer agents could fail *in vitro*, while *in silico* analysis of active metabolites would correctly identify efficacy. Thus, given its relative low cost to other methods, and effectiveness in identifying strong therapeutic candidates, *in silico* technologies are highly suited for repurposing initiatives that could potentially yield novel FDA- and EMA-approved medicines.

## Considerations in Regulatory Affairs and Intellectual Property (IP)

### Regulatory Approval Pathways

In the US, there are three separate regulatory approval pathways that allow for the registration of distinct classifications of drugs, as outlined in the Food, Drug and Cosmetics Act,^6^ although only one of these [i.e., “505(b)(2)”], is relevant to drug repurposing. All drug candidates for repurposing must be submitted through Section 505(b)(2), regardless of whether it is for cancer therapeutics or alternate diseases. Section 505(b)(2) became available in 1984 under the Drug Price Competition and Patent Term Restoration Act (Hatch-Waxman Amendments),^7^ but only recently have applications to this pathway grown in popularity. Data show that approximately twice as many products receive FDA approval through 505(b)(2) than the novel drug route [i.e. “505(b)(1)”],^8^^,^^9^ suggesting that companies are looking to generate new revenue and exclusivity from short approval timelines.

The 505(b)(2) pathway allows for the registration of a drug for which at least one of the studies relied upon for approval was not conducted by the applicant.^10^ Thus, applicants can partially rely on published literature and the FDA’s previous findings regarding safety of an approved product to supplement their data. Accordingly, fewer supporting studies are required, resulting in shorter timelines and reduced costs. Furthermore, to achieve the 505(b)(2) approval, drug developers must identify a unique administration route or disease indication for their repurposed drug compared with the primary route and indication.^11^

In Europe, a parallel approval pathway is regulated by the EMA under Article 10 of Directive 2001/83/EC. However, in contrast to section 505(b)(2) of the Food, Drug and Cosmetics Act, which allows the use of non-proprietary studies that have previously achieved a high standard of quality and safety to support any part of an application, Article 10 concerns drugs that require studies tailored to the differences from reference listed drugs—it does not provide a legal basis for the use of non-proprietary studies.^12^ Another important difference is that Article 10 cannot be used for new molecular entities as, by definition, only changes from the reference listed drug apply. On average, it takes the EMA 6 months longer than the FDA to approve new indications for a drug.

## Combining IP and Regulatory Exclusivities for Commercial Success

To commercialize a repurposed drug, it is important to consider factors in IP and regulatory exclusivities. For example, a repurposed drug can affect the market exclusivity of original claims. Between previously abandoned/unapproved drug products [i.e., shelved active pharmaceutical ingredients (APIs)], and new formulations/indications for existing marketed drugs, shelved APIs provide an attractive opportunity since they offer excellent product protection (29).^13^ Further, repurposing an approved API for a secondary indication without reformulation would only benefit the original manufacturer as they own the IP for the asset; thus, drug makers could generate additional revenue from new markets. However, by modifying the formulation of a repurposed drug, inventors unassociated with the original manufacturer could be granted novel IP since the invention would be deemed as a new composition of matter. Therefore, the best protection is provided by patents which safeguard the composition of the API (29).^14^ However, such patents are filed early in the drug development process, making patent life short-lived once the product is ready for market. Alternatively, new composition of matter patents is available when the repurposed API incorporates a new formulation. These patents may be eligible for a 5-year extension to compensate for the time lost during the drug approval process (see text footnote 7).^15^

Although a robust patent protects against competitors, the regulatory exclusivities provided by the Hatch-Waxman Act may also provide ample protection. Even when patent protection is unavailable, the duration of market exclusivity may provide suitable time to profit from initial investments. Further, new chemical entity exclusivity is granted if the drug product has an API not yet registered by the FDA, regardless of the development duration.^16^^,^^17^ This will prevent other companies from relying on the approved drug’s safety and efficacy data for at least 5 years (30), and also prevents the FDA from accepting applications for generic versions within the first 4 years of the exclusivity period. The EMA, in contrast, will grant up to 8 years of exclusivity. In addition, and of interest to cancer drug development, the US Orphan Drug Act incentivizes drug makers to repurpose drugs for rare malignancies by providing a 7-year market exclusivity and a 50% tax credit for expenditures incurred during the clinical testing phase (see text footnote 15)^18^; the recently passed Orphan Product Extensions Now Accelerating Cures and Treatments Act will extend this exclusivity by 6 months.^19^ If an anticancer drug benefits pediatric populations, an additional 6 months may also be granted (see text footnote 15). A similar orphan medicine designation exists in Europe, offering 10-year market protection and reduced fees for regulatory activities.^20^ If the orphan drug is specific to pediatric populations, 12-year market protection will be applied.^21^

Overall, when a drug developer selects an existing therapeutic for repurposing with a focus on cancer, consideration must be given to IP and market exclusivity rights which will influence the drug’s success. The greatest degree of protection is associated with APIs that have not previously been on the market or approved by the FDA or EMA. Substantial protection can also be achieved with strategic use of new composition of matter patents, allowing for comprehensive security in the marketplace well past regulatory approval.

## Commercial Success of Repurposed Drugs in Cancer

Despite significant increases in spending over the past few decades, pharmaceutical companies have had fewer drugs approved than ever before (31). This trend, termed “Eroom’s Law,” describes the inverse correlation of large monetary input into drug development and the deceleration of drug discovery (32). Consequently, repurposing of existing drugs is frequently explored as a strategy to correct this trend. According to BCC Research, the global market for drug repurposing reached $24.4 billion in 2015 and is projected to reach $31.3 billion by 2020.^22^ Further, pharmaceutical companies are already investigating the ability to repurpose drugs for various cancers. For example, the manufacturers of metformin (a diabetes treatment) and celecoxib (anti-inflammatory) are testing the efficacy of these drugs on patients with breast and colon cancer within several clinical trials^23^ (1).

However, when repurposing existing compounds to increase their profitability, there are several factors that must be considered (33). For example, companies must be wary of patent expiry dates of existing compounds; when a patent expires, others can produce generic versions at lower costs. This was evident in the case of celebrex (developed by Pfizer), such that when its patent expired in 2014, Teva Pharmaceuticals released a generic version and Pfizer experienced nearly a 10% loss of operational revenue in the first quarter of 2015.^24^ Therefore, it is imperative that drug developers apply strategies that have previously been successful (e.g., reformulation and off-label usage), to bring newly repurposed anticancer agents to market (33). Otherwise, such financial disincentives (i.e., patent expiration, and other policies, rules, or taxes that discourage or prevent further development) could greatly hinder global drug repurposing efforts.

## Strategies Implemented in the Industry

A key to maximizing commercial value is to secure ownership (i.e., licensing), or if already owned, extending its patent life cycle (34, 35). Interestingly, many companies (e.g., Sosei Co. Ltd.) specialize in acquiring and subsequently out-licensing drug libraries to organizations looking to establish drug pipelines (5, 36). In the US, this approach has reportedly increased the value of such assets by ~10% on average annually (37). Another approach which has been implemented consists of targeting specific indications, namely orphan diseases. These are defined in the US as affecting less than 200,000 people^25^^,^^26^ and can include rare cancers such as Ewing sarcoma (38), adrenocortical carcinoma (39), gastrointestinal stromal tumors (40), and chordomas (41). Given the lack of competitive pressure and available treatments for these diseases, approval of potential therapies tends to be fast-tracked by regulatory authorities (42).

Most opportunities aimed at producing affordably priced drugs for orphan diseases mainly reside in drug repurposing (43). However, there is also potential for the opposite, such that drug companies usually price these therapies at a substantially greater value than a middle-class individual could afford, and insurance companies typically cover only a portion of the cost of treatments for such rare diseases (44). In addition, several controversies exist regarding motives behind drug repurposing endeavors. For example, affordable medicines can become unaffordable once a company discovers that it can be used to treat a less common indication; this type of “price gouging,” where prices of inexpensive products skyrocket seemingly overnight under conditions of corporate monopoly, does not benefit patients with such unmet medical needs (43, 45). Accordingly, lawmakers are currently determining how to prevent such events from becoming standard practice (45). Further, it is predicted that by 2020, a fifth of drug sales will come from orphan disease treatments including those for various cancers,^27^ representing a partial shift away from common illnesses such as diabetes, asthma or cardiovascular diseases, and therefore, a balance between price and profit must be achieved to maintain drug affordability for both patients and drug makers alike (8). By incentivizing drug makers to invest years and billions of dollars into drug development for rare and orphan diseases *via* various mechanisms (e.g., subsidies, tax credits, and fast-tracked drug approval), patients with rare conditions benefit as they can expect less expensive medicines than if such incentives were not in place.

Once a new drug has been approved for a new use, a typical strategy to maximize profit is to obtain a “specialty drug” designation so that it can only be sold in specialty pharmacies. This labeling results in a price mark-up, since these pharmacies require more operational funding to store and handle these treatments, as well as results in a prolonging of patent exclusivity (46). This approach has been employed previously for several anticancer drugs, including rituxan (a non-Hodgkin’s lymphoma and chronic myeloid leukemia therapy) which, under its specialty drug designation, is predicted to become the second most profitable orphan disease therapy by 2022 (see text footnote 23).

Finally, it is important to note that following the discovery of a drug that can be repurposed as a cancer therapy, many academics and companies opt to create partnerships with appropriate pharmaceutical leaders in the oncology therapeutics industry (47). This can be seen in repurposing-centric companies such as BioVista establishing collaborations with Pfizer and Novartis, or NuMedii with Astellas Pharmaceuticals.^28^^,^^29^ By aligning with larger, well-established organizations, additional resources and capital become available to facilitate translational research and entry of a drug into the repurposing pipeline (48), including functional validation studies and ultimately clinical trials.

## Conclusion and Perspectives

**Figure 1:**
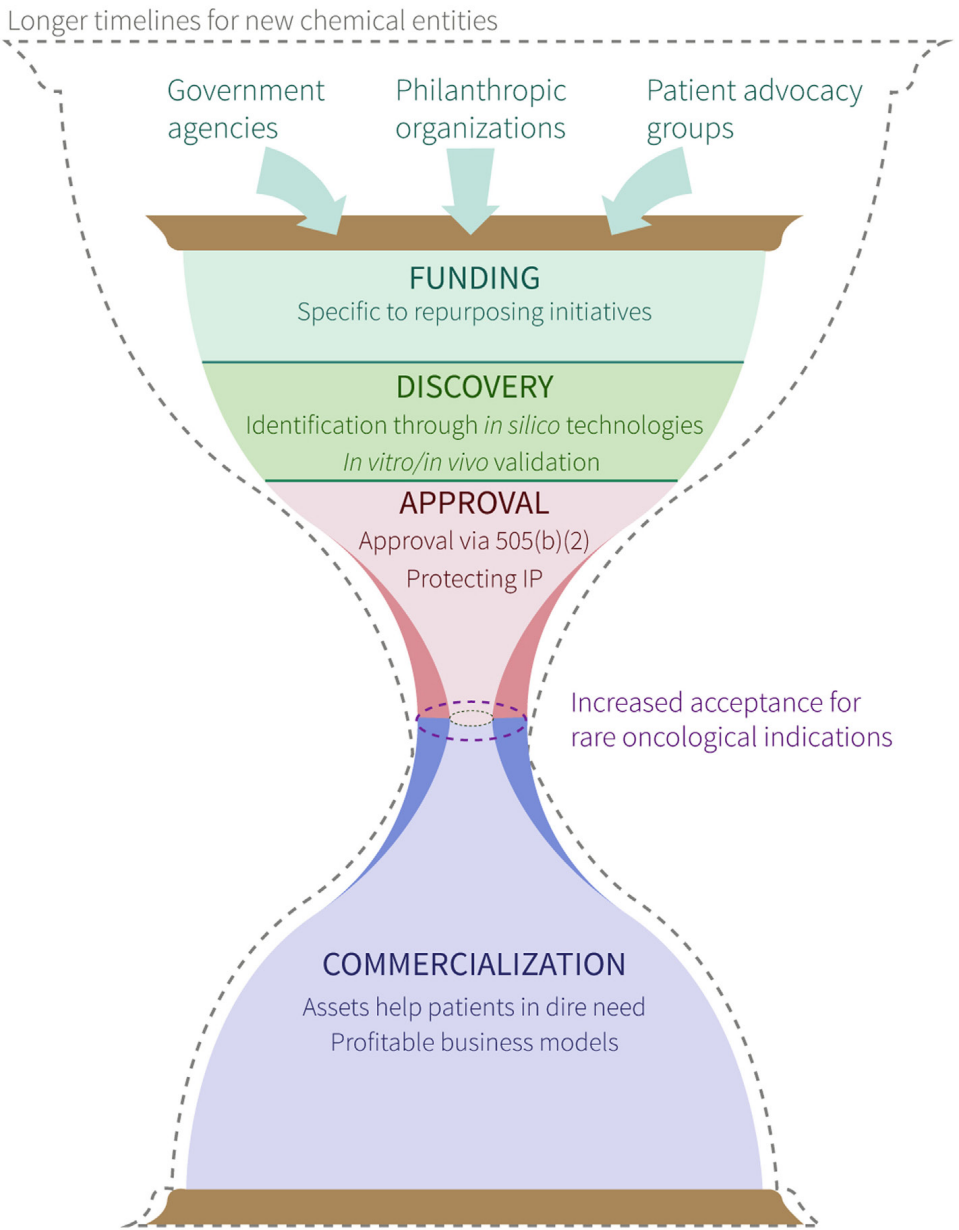
Overview of process to repurpose existing drugs for cancer indications. Symbolic (i.e., hourglass) representation of a proposed timeline for new chemical entities (dashed hourglass outline) and repurposed drugs (solid, colored hourglass) from hypothesis generation and project funding, to product commercialization and patient benefit. Labels within funding, discovery, approval, and commercialization stages denote requirements/strategies for successful movement through each. The center of the hourglass denotes the “bottleneck” between approval and commercialization, and highlights the facilitated approval of drugs for rare oncological indications (purple dashed oval) compared with drugs repurposed for other indications (gray dashed oval).

Although careful considerations must be made with respect to the process of navigating the complex ecosystems of medical regulations and commercialization procedures (Figure 1), drug repurposing for cancer indications has the potential to impact a significant number of patients in which there is currently an urgent medical need. It is important to note that when repurposed therapies demonstrate improved efficacy, safety and/or cost over the current standard(s) of care, both patients and drug developers alike reap the benefits. Pharmaceutical companies can save both time and money in drug development by streamlining validation studies without the need to reproduce in-human safety studies, whereas patients gain access to novel, fast-tracked approaches aimed at treating their personalized disease. Furthermore, as new technologies emerge throughout the current “-omics era,” biological multi-systems-level big data will continue to be leveraged to facilitate additional successes in the repurposing of approved, investigational and potentially even hypothetical (i.e., yet to be synthesized) drugs for multiple indications in oncology.

## Author Contributions

The manuscript was primarily written by JH, MP, LS, and CY with input from all other authors.

## Conflict of Interest Statement

All authors affiliated with Cyclica are employees of the company. NK, VS, and SM were employed by Cyclica Inc. during the course of the study and are shareholders of the company. All other authors declare that they have no competing interests.

## Footnotes

^1^“Treatment for Endogenous Cushing’s Syndrome”: https://clinicaltrials.gov/ct2/show/NCT01838551 [last accessed June 15, 2017].

^2^“Funding and Notices”: https://ncats.nih.gov/funding [last accessed June 11, 2017].

^3^“Rare Diseases Drug Repurposing Funding Opportunities”: http://www.cihr-irsc.gc.ca/e/49739.html [last accessed June 10, 2017].

^4^“Research in Progress”: http://www.cureswithinreach.org/research/search-complete-research/research-projects [last accessed June 11, 2017].

^5^“Projects”: http://www.anticancerfund.org/projects/currently-ongoing [last accessed June 12, 2017].

^6^21 U.S.C. § 301.

^7^21 U.S.C. § 355 and § 360cc.

^8^21 U.S.C. § 355(b)(1).

^9^“An increasing number of companies are using a once-obscure FDA drug approval pathway”: Regulatory Focus, http://www.raps.org/Regulatory-Focus/News/2015/04/08/21933/An-Increasing-Number-of-Companies-Are-Using-a-Once-Obscure-FDA-Drug-Approval-Pathway/ [last accessed June 16, 2017].

^10^21 U.S.C. § 355(b)(2).

^11^“Guidance for Industry: Applications Covered by Section 505(b)(2)”: United States Department of Health and Human Services, Center for Drug Evaluation and Research, https://www.fda.gov/downloads/Drugs/Guidances/ucm079345.pdf [last accessed June 19, 2017].

^12^Article 10 of Directive 2001/83/EC as amended.

^13^Barratt M, Frail D, editors. *Drug Repositioning: Bringing New Life to Shelved Assets and Existing Drugs*. Hoboken: John Wiley & Sons (2012).

^14^Ellery T, Hansen N. *Pharmaceutical Lifecycle Management: Making the Most of Each and Every Brand*. Hoboken: John Wiley & Sons (2012).

^15^21 CFR § 60 (1984).

^16^“Patents and Exclusivity”: FDA/CDER SBIA Chronicles, https://www.fda.gov/downloads/drugs/developmentapprovalprocess/smallbusinessassistance/ucm447307.pdf [last accessed June 19, 2017].

^17^21 C.F.R. § 314.108 (1999).

^18^21 C.F.R. § 316 (1992).

^19^H.R. 1223.

^20^Article 8(1) of Regulation EC/141/2000.

^21^Article 37 of Regulation EC/1901/2006.

^22^Dewan SS. Global Markets for Drug Repurposing (2016). Retrieved from https://www.bccresearch.com/market-research/pharmaceuticals/drug-repurposing-markets-report-phm175a.html.

^23^https://clinicaltrials.gov/ct2/show/NCT00045591 [last accessed June 28, 2017].

^24^“Pfizer Reports First-Quarter 2015 Results”: https://s21.q4cdn.com/317678438/files/doc_news/2015/Q1_2015_PFE_Earnings_Press_Release_foijsdflskd.pdf [last accessed June 25, 2017]

^25^42 U.S.C. § 201 and § 283h.

^26^21 C.F.R. § 316.21 (2012).

^27^“EvaluatePharma Orphan Drug Report 2017”: http://info.evaluategroup.com/rs/607-YGS-364/images/EPOD17.pdf [last accessed May 24, 2017].

^28^“Collaborations”: https://www.biovista.com/about/collaborations/ [last accessed June 27, 2017].

^29^“New Indications Discovery Collaboration”: http://numedii.com/numedii-announces-new-indications-discovery-collaboration-with-astellas/ [last accessed June 27, 2017].

## References

[1] Kasznicki J Sliwinska A Drzewoski J Metformin in cancer prevention and therapy. Ann Transl Med (2014) 2:57 10.3978/j.issn.2305-5839.2014.06.01 25333032 PMC4200668

[2] Grunberg SM Weiss MH Russell CA Spitz IM Ahmadi J Sadun A Long-term administration of mifepristone (RU486): clinical tolerance during extended treatment of meningioma. Cancer Invest (2006) 24(8):727–33. 10.1080/07357900601062339 17162554

[3] Telleria C Drug repurposing for cancer therapy. J Cancer Sci Ther (2012) 4:ix–xi. 10.4172/1948-5956.1000e108 22984635 PMC3440183

[4] Pantziarka P Bouche G Meheus L Sukhatme V Sukhatme VP Vikas P. The Repurposing Drugs in Oncology (ReDO) Project. Ecancermedicalscience (2014) 8:442. 10.3332/ecancer.2014.442 25075216 PMC4096030

[5] Ashburn TT Thor KB Drug repositioning: identifying and developing new uses for existing drugs. Nat Rev Drug Discov (2004) 3(8):673–83. 10.1038/nrd1468 15286734

[6] Chong CR Sullivan DJ New uses for old drugs. Nature (2007) 448(7154):645–6. 10.1038/448645a 17687303

[7] Oprea TI Overington JP. Computational and practical aspects of drug repositioning. Assay Drug Dev Technol (2015) 13:299–306. 10.1089/adt.2015.29011.tiodrrr 26241209 PMC4533090

[8] Bertolini F Sukhatme VP Bouche G Drug repurposing in oncology – patient and health systems opportunities. Nat Rev Clin Oncol (2015) 12(12):732–42. 10.1038/nrclinonc.2015.169 26483297

[9] Pantziarka P Scientific advice – is drug repurposing missing a trick? Nat Rev Clin Oncol (2017) 14(8):455–6. 10.1038/nrclinonc.2017.69 28534529

[10] Qu XA Rajpal DK. Applications of Connectivity Map in drug discovery and development. Drug Discov Today (2012) 17:1289–98. 10.1016/j.drudis.2012.07.017 22889966

[11] Li J Zheng S Chen B Butte AJ Swamidass SJ Lu Z. A survey of current trends in computational drug repositioning. Brief Bioinform (2016) 17:2–12. 10.1093/bib/bbv020 25832646 PMC4719067

[12] Lamb J Crawford E Peck D Modell J Blat I Wrobel M The connectivity map: using gene-expression signatures to connect small molecules, genes, and disease. Science (2006) 313:1929–35. 10.1126/science.1132939 17008526

[13] Marques LO Lima MS Soares BG. Trifluoperazine for schizophrenia. Cochrane Database Syst Rev (2004) 1:CD003545. 10.1002/14651858.CD003545 14974020 PMC7003674

[14] Yeh C-TT Wu AT Chang PM Chen K-YY Yang C-NN Yang S-CC Trifluoperazine, an antipsychotic agent, inhibits cancer stem cell growth and overcomes drug resistance of lung cancer. Am J Respir Crit Care Med (2012) 186:1180–8. 10.1164/rccm.201207-1180OC 23024022

[15] Chen M-H Yang W-L Lin K-T Liu C-H Liu Y-W Huang K-W Gene expression-based chemical genomics identifies potential therapeutic drugs in hepatocellular carcinoma. PLoS One (2011) 6:e2718. 10.1371/journal.pone.0027186 22087264 PMC3210146

[16] Huang L Zhao S Frasor J Dai Y. An integrated bioinformatics approach identifies elevated cyclin E2 expression and E2F activity as distinct features of tamoxifen resistant breast tumors. PLoS One (2011) 6:e22274. 10.1371/journal.pone.0022274 21789246 PMC3137633

[17] Lee BK Tiong KH Chang JK Liew CS Abdul Rahman ZA Tan AC DeSigN: connecting gene expression with therapeutics for drug repurposing and development. BMC Genomics (2017) 18:934. 10.1186/s12864-016-3260-7 28198666 PMC5310278

[18] Ke K Li H Yao H Shi X Dong C Zhu Y In silico prediction and in vitro and in vivo validation of acaricide fluazuron as a potential inhibitor of FGFR3 and a candidate anticancer drug for bladder carcinoma. Chem Biol Drug Des (2017) 89:505–13. 10.1111/cbdd.12872 27664399

[19] Mihara M Shintani S Nakahara Y Kiyota A Ueyama Y Matsumura T Overexpression of CDK2 is a prognostic indicator of oral cancer progression. Jpn J Cancer Res (2001) 92:352–60. 10.1111/j.1349-7006.2001.tb01102.x 11267947 PMC5926707

[20] Kim SJ Nakayama S Miyoshi Y Taguchi T Tamaki Y Matsushima T Determination of the specific activity of CDK1 and CDK2 as a novel prognostic indicator for early breast cancer. Ann Oncol (2008) 19:68–72. 10.1093/annonc/mdm358 17956886

[21] Hongo F Takaha N Oishi M Ueda T Nakamura T Naitoh Y CDK1 and CDK2 activity is a strong predictor of renal cell carcinoma recurrence. Urol Oncol (2014) 32:1240–6. 10.1016/j.urolonc.2014.05.006 25443276

[22] Chohan T Qian H Pan Y Chen J-Z Cyclin-dependent kinase-2 as a target for cancer therapy: progress in the development of CDK2 inhibitors as anti-cancer agents. Curr Med Chem (2014) 22:237–63. 10.2174/0929867321666141106113633 25386824

[23] Huang C-Y Huang C-H Chang P Wu M-Y Ng K-L. In silico identification of potential targets and drugs for non-small cell lung cancer. IET Syst Biol (2014) 8:56–66. 10.1049/iet-syb.2013.0035 25014226 PMC8687210

[24] Shi X-NN Li H Yao H Liu X Li L Leung K-SS Adapalene inhibits the activity of cyclin-dependent kinase 2 in colorectal carcinoma. Mol Med Rep (2015) 12:6501–8. 10.3892/mmr.2015.4310 26398439 PMC4626183

[25] Shi X-NN Li H Yao H Liu X Li L Leung K-SS In silico identification and in vitro and in vivo validation of anti-psychotic drug fluspirilene as a potential CDK2 inhibitor and a candidate anti-cancer drug. PLoS One (2015) 10:e0132072. 10.1371/journal.pone.0132072 26147897 PMC4493148

[26] Lan M-Y Chen C-L Lin K-T Lee S-A Yang W-L Hsu C-N From NPC therapeutic target identification to potential treatment strategy. Mol Cancer Ther (2010) 9:2511–23. 10.1158/1535-7163.MCT-09-0966 20716640

[27] Lan M Yang W Lin K Lin J Shann Y Ho C Using computational strategies to predict potential drugs for nasopharyngeal carcinoma. Head Neck (2014) 36:1398–407. 10.1002/hed.23464 24038431

[28] Desta Z Ward BA Soukhova NV Flockhart DA. Comprehensive evaluation of tamoxifen sequential biotransformation by the human cytochrome P450 system in vitro: prominent roles for CYP3A and CYP2D6. J Pharmacol Exp Ther (2004) 310:1062–75. 10.1124/jpet.104.065607 15159443

[29] Smith RB Repositioned drugs: integrating intellectual property and regulatory strategies. Drug Discov Today Ther Strateg (2011) 8:131–7. 10.1016/j.ddstr.2011.06.008

[30] Prajapati V Tripathy S Dureja H Product lifecycle management through patents and regulatory strategies. J Med Market (2013) 13:171–80. 10.1177/1745790413497388

[31] Munos B Lessons from 60-years of pharmaceutical innovation. Nat Rev Drug Discov (2009) 8:959–68. 10.1038/nrd2961 19949401

[32] Scannell JW Blanckey A Boldon H Warrington B Diagnosing the decline in pharmaceutical R&D efficiency. Nat Rev Drug Discov (2012) 11(3):191–200. 10.1038/nrd3681 22378269

[33] Verbaanderd C Meheus L Huys I Pantziarka P. Repurposing drugs in oncology: next steps. Trends Cancer (2017) 3(8):543–6. 10.1016/j.trecan.2017.06.007 28780930

[34] Lu Y Penrod JR Sood N Woody S Phillipson T Dynamic cost-effectiveness of oncology drugs. Am J Manag Car (2012) 11(Suppl):S249–56. 23327456

[35] Berger J Dunn JD Johnson MM Karst KR Shear WC How drug life-cycle management patent strategies may impact formulary management. Am J Manag Care (2017) 22(16):S487–95. 28719222

[36] Stuart M Sosei’s drug re-profiling strategy: a three-way street. Start-Up (2003) 21:6.

[37] Savage P Mahmoud S Patel Y Kantarijan H. Cancer drugs: an international comparison of postlicensing price inflation. J Oncol Pract (2017) 13(6):e538–42. 10.1200/JOP.2016.014431 28605615

[38] Passetto ZY Chen B Alturkmani H Hyter S Flynn CA Baltezor M In silico and in vitro drug screening identifies new therapeutic approaches for Ewing sarcoma. Oncotarget (2017) 8(3):4079–95. 10.18632/oncotarget.13385 27863422 PMC5354814

[39] Satoh K Zhang L Zhang Y Chelluri R Boufraqech M Nilubol N Identification of niclosamide as a novel anticancer agent for adrenocortical carcinoma. Clin Cancer Res (2016) 22(14):3458–66. 10.1158/1078-0432.CCR-15-2256 26873959 PMC4947455

[40] Passetto ZY Ma Y Hirst JJ von Mehren M Weir SJ Godwin AK. Drug repurposing identifies a synergistic combination therapy with imatinib mesylate for gastrointestinal stromal tumor. Mol Cancer Ther (2014) 13(10):2276–87. 10.1158/1535-7163.MCT-14-0043 25122069 PMC4185217

[41] Xia M Huang R Sakamuru S Alcorta D Cho MH Lee DH Identification of repurposed small molecule drugs for chordoma therapy. Cancer Biol Ther (2013) 14(7):638–47. 10.4161/cbt.24596 23792643 PMC3742493

[42] Maxmen A Busting the billion-dollar myth: how to slash the cost of drug development. Nature (2016) 536:388–90. 10.1038/536388a 27558048

[43] Davies EH Fulton E Brook D Hughes DA. Affordable orphan drugs: a role for not-for-profit organizations. Br J Clin Pharmacol (2017) 83(7):1595–601. 10.1111/bcp.13240 28109021 PMC5465340

[44] Handfield R Feldstein J Insurance companies’ perspectives on the orphan drug pipeline. Am Health Drug Benefits (2013) 6(9):589–98. 24991385 PMC4046481

[45] Houston AR Beall RF Attaran A. Upstream solutions for price-gouging on critical generic medicines. J Pharm Policy Pract (2016) 9:15. 10.1186/s40545-016-0064-8 27141308 PMC4852412

[46] Gleason PP Alexander GC Starner CI Ritter ST Van Houten HK Gunderson BW Health plan utilization and costs of specialty drugs within 4 chronic conditions. J Manag Care Pharm (2013) 19(7):542–8. 10.18553/jmcp.2013.19.7.542 23964615 PMC10437312

[47] Oprea TI Bauman JE Bologa CG Buranda T Chigaev A Edwards BS Drug repurposing from an academic perspective. Drug Discov Today Ther Strateg (2011) 8(3–4):61–9. 10.1016/j.ddstr.2011.10.002 22368688 PMC3285382

[48] Yildirim O Gottwald M Schüler P Michel MC. Opportunities and challenges for drug development: public-private partnerships, adaptive designs and big data. Front Pharmacol (2016) 7:461. 10.3389/fphar.2016.00461 27999543 PMC5138214

